# Associations between the gut microbiota and host immune markers in pediatric multiple sclerosis and controls

**DOI:** 10.1186/s12883-016-0703-3

**Published:** 2016-09-21

**Authors:** Helen Tremlett, Douglas W. Fadrosh, Ali A. Faruqi, Janace Hart, Shelly Roalstad, Jennifer Graves, Collin M. Spencer, Susan V. Lynch, Scott S. Zamvil, Emmanuelle Waubant, US Network of Pediatric MS Centers, Greg Aaen, Anita Belman, Leslie Benson, Charlie Casper, Tanuja Chitnis, Mark Gorman, Yolanda Harris, Lauren Krupp, Tim E. Lotze, Sabeen Lulu, Jayne Ness, Cody Olsen, Erik Roan, Moses Rodriguez, John Rose, Timothy C. Simmons, Jan-Mendelt Tillema, Wendy Weber, Bianca Weinstock-Guttman

**Affiliations:** 1 Faculty of Medicine (Neurology), University of British Columbia, Room S178, 2211 Wesbrook Mall, Vancouver, BC V6T 2B5 Canada; 2 University of California, San Francisco, CA USA; 3 University of Utah, Salt Lake City, UT USA

**Keywords:** Pediatric multiple sclerosis, Gut microbiota, 16S rRNA, Case–control study, Risk factors, Immune markers, Disease-modifying drugs, Microbiota-immune balance

## Abstract

**Background:**

As little is known of association(s) between gut microbiota profiles and host immunological markers, we explored these in children with and without multiple sclerosis (MS).

**Methods:**

Children ≤18 years provided stool and blood. MS cases were within 2-years of onset. Fecal 16S rRNA gene profiles were generated on an Illumina Miseq platform. Peripheral blood mononuclear cells were isolated, and Treg (CD4^+^CD25^hi^CD127^low^FoxP3^+^) frequency and CD4^+^ T-cell intracellular cytokine production evaluated by flow cytometry. Associations between microbiota diversity, phylum-level abundances and immune markers were explored using Pearson’s correlation and adjusted linear regression.

**Results:**

Twenty-four children (15 relapsing-remitting, nine controls), averaging 12.6 years were included. Seven were on a disease-modifying drug (DMD) at sample collection. Although immune markers (e.g. Th2, Th17, Tregs) did not differ between cases and controls (*p* > 0.05), divergent gut microbiota associations occurred; richness correlated positively with Th17 for cases (*r* = +0.665, *p* = 0.018), not controls (*r* = −0.644, *p* = 0.061). *Bacteroidetes* inversely associated with Th17 for cases (*r* = −0.719, *p* = 0.008), not controls (*r* = +0.320, *p* = 0.401). *Fusobacteria* correlated with Tregs for controls (*r* = +0.829, *p* = 0.006), not cases (*r* = −0.069, *p* = 0.808).

**Conclusions:**

Our observations motivate further exploration to understand disruption of the microbiota-immune balance so early in the MS course.

**Electronic supplementary material:**

The online version of this article (doi:10.1186/s12883-016-0703-3) contains supplementary material, which is available to authorized users.

## Background

Multiple sclerosis (MS) is thought to be an autoimmune disease in which components of the immune system target cells in the brain and spinal cord, resulting in demyelination and axonal damage. While the cause(s) are unknown, both genetic and early life environmental exposures are implicated. Emerging studies have shown perturbations in the gut microbiota of individuals with MS relative to controls [5, 22, 27]. The gut microbiota’s role in modulating the host’s immune system could be highly relevant for MS. However, unlike the well-studied relationship between the immune system and MS disease processes, little is known of the gut microbiota-immune relationship in MS. The gut microbiota appears highly influential in stimulating a pro-inflammatory T cell response and subsequent disease in animal models of MS [4, 17]. Further, perturbations to the gut microbiota composition (dysbiosis) have been linked to other immune-mediated diseases distal from the gut, including rheumatoid arthritis, type 1 diabetes, atopic dermatitis and asthma [20]. These conditions could all have their ‘pathogenic origins’ in the immune response modulation by the microbiota [20].

Compared to adult MS, pediatric MS represents a unique opportunity to examine such associations close to the original exposures and biological onset of disease. Further, children have had a limited lifetime of exposures, and hence fewer potential confounding effects. We conducted a pilot study to explore the association(s) between the gut microbiota and blood immunological markers in disease-modifying drug (DMD) naïve and exposed pediatric MS cases early in their disease course and healthy controls.

## Methods

Children ≤18 years old accessing a general or MS specific pediatric clinic at the University of California, San Francisco (UCSF), USA were invited to participate in an environmental risk factor study; those providing both a stool and blood sample formed the current study cohort. MS cases had <2 years of disease (from symptom onset). Controls were similar for age and sex, were not known to have any autoimmune disorders (except for asthma or eczema) and had neither parent diagnosed with MS nor a related disorder. No child was exposed to a systemic antibiotic in the 2 months prior to stool collection. Cohort characteristics were obtained from participants via questionnaires, and standardized forms completed by the physician or research coordinator (JH) supported by chart abstraction, as described previously [27]. The UCSF Institutional Review Board approved the study. All subjects and a parent signed an assent/consent form.

### Stool collection, DNA extraction and 16S rRNA profiling

Participants were asked to collect and ship overnight (on ice) a sample of the child’s first stool of the day to UCSF where it was stored at −80 °C. DNA was then extracted from approximately 25 mg of stool using PowerSoil® DNA Isolation Kit (MO BIO Laboratories, Inc, Carlsbad, CA) prior to amplification of the V4 hypervariable region of the bacterial 16S rRNA gene was amplified in triplicate [7]. Amplicons were combined, purified and pooled in equimolar concentrations and sequenced using a Miseq platform (Illumina, Inc., San Diego, CA) with a 251×151 base pair run. The 16S rRNA reads were grouped using a ≥97 % similarity threshold into operational taxonomic units (OTUs, i.e., ‘taxa’), which were singly rarefied to 201,546 reads per sample. Taxonomy was assigned using the Greengenes database via the QIIME (Quantitative Insights Into Microbial Ecology) [6, 9] platform. Community alpha diversity was expressed as richness (the number of unique taxa identified); evenness (taxon distribution), and Faith’s phylogenic diversity metric (which weights phylogenetic (i.e. evolutionary) relationships between taxa) [8], using the *Vegan* and *Picante* packages (R software [24]), Additional file 1: Appendix 1, supplementary methods.

### Blood immune markers

Peripheral blood mononuclear cells (PBMCs) were isolated by density gradient separation (Ficoll-Paque, GE Healthcare), and cryopreserved on the day of collection. Thawed PBMCs were allowed to recover in culture overnight, and then plated at 1.5 × 10^6^ cells/ml in X-VIVO 15 media (ThermoFisher), supplemented with 5 % human AB serum (Life Technologies). Cells were stimulated with PMA (50 ng/ml) + ionomycin (0.5 μg/ml), or media alone, for 5 h in the presence of brefeldin A (GolgiPlug, BD Biosciences). Treg frequency and intracellular cytokine production by T cells were evaluated by flow cytometry. Treg (CD4^+^CD25^hi^CD127^low^FoxP3^+^) frequency was expressed as a proportion of total CD3^+^ T cells. Frequencies of the following CD4^+^ T cell subsets were quantitated as: Th1 (IFN-γ^+^), Th17 (IL-17^+^), Th2 (IL-4^+^), and Tr1 (IL-10^+^). Total (CD3^+^) T cells and CD4^+^ T cells were reported as proportions of PBMC and total T cell populations, respectively.

### Statistical analyses

Cohort characteristics were presented descriptively. Blood immune markers were compared between controls and cases (all cases, then DMD naïve and exposed separately) using non-parametric tests (Mann–Whitney and Kruskal-Wallis tests) and associations with the gut microbiota metrics (alpha diversity and phylum-level abundances) were initially explored via Pearson’s correlation coefficient. Where associations were at least ‘modest [12]’ and significant (absolute *r* > 0.5 and *p* < 0.05), linear regression analyses were performed, with the gut microbiota metrics the independent and immune markers the dependent variables, adjusting for age, disease duration, DMD exposure or case or control status, as appropriate (Additional file 1: Appendix 1). Adjusting factors were selected based on clinical relevance (i.e., disease duration, DMD exposure) and/or the limited pediatric literature which indicated that age [13] and DMD exposure [27] could influence the relationship between the developing gut microbiota and immune system. Due to the small sample size, exploration of relationships at the taxon level was not performed for this study. No corrections were applied for multiple testing, although the expected number of chance findings from the Pearson’s correlations performed were estimated as follows: seven immune markers were assessed for correlation with three diversity metrics and 13 phyla, resulting in 7×3 = 21 tests and 7×13 = 91 tests, respectively. Chance significance would be expected for one in 20 tests, i.e. approximately one test (diversity) and 4–5 tests (phylum-level). Statistical analyses were performed using the Statistical Package for the Social Sciences (SPSS for Windows, Ver. 22.0. Armonk, NY: IBM Corp. 2013).

## Results

Twenty-four children (15 relapsing-remitting MS cases, nine controls) with a mean age of 12.6 years (SD = 4.18; range 4–18) fulfilled criteria and provided both stool and blood samples; 9/24 (38 %) were boys. All cases met McDonald criteria, had relapsing-remitting MS and a short disease duration (mean = 10.0 months; range 2–23 months at stool collection). The mean age at MS onset was 11.5 years (SD = 4.84; range 4–17); seven (47 %) MS cases were exposed to a DMD and five (33 %) a corticosteroid (in the 2 months pre-stool sample). Although more controls self-identified as white (67 % controls, 33 % cases) and controls averaged almost 2 years older than cases, the proportions over 12 years were rather similar (56 % and 60 % respectively) and no characteristic (Table 1) differed significantly between cases and controls (all *p* > 0.2, not shown). Additional demographic, lifestyle and clinical characteristics are shown in Table 1 and Additional file 1: eTables 1.1 and 1.2, including the annualized relapse rate (mean = 0.91; SD = 0.851), time since last relapse (mean = 181.1 days; SD = 142.17) and dietary metrics (fat/fibre groupings) which were similarly distributed between cases and controls. All stool samples were collected within 3 months of the blood draw (median = 13.5 days; range 0–85 days). Most stool samples (*n* = 21) were collected after the blood draw, two before and one on the same day.

**Table 1:** Characteristics of the pediatric multiple sclerosis (MS) cases and controls

Characteristic, n (%) unless stated otherwise	MS cases, *n* = 15	Controls, *n* = 9	Cases and controls, *n* = 24
Sex			
Girl	8 (53 %)	7 (78 %)	15 (63 %)
Boy	7 (47 %)	2 (22 %)	9 (38 %)
Age at stool sample collection, years: mean (SD; range)	11.9 years (SD = 4.64; 4–17)	13.8 years (SD = 3.19; 9–18)	12.6 years (SD = 4.18; 4–18)
Age at stool sample collection			
≤ 12 years old	6 (40 %)	4 (44 %)	10 (42 %)
> 12 years old	9 (60 %)	5 (56 %)	14 (58 %)
Race			
White	5 (33 %)	6 (67 %)	11 (46 %)
Non-white	10 (67 %)	3 (33 %)	13 (54 %)
Ethnicity			
Hispanic	6 (40 %)	3 (33 %)	9 (38 %)
Non- Hispanic	9 (60 %)	6 (67 %)	15 (63 %)
Co-morbid condition^a^			
Present	6 (40 %)	2 (22 %)	8 (33 %)
Absent	9 (60 %)	7 (78 %)	16 (67 %)

Key: *SD* standard deviation

^a^Comorbid conditions were collected pre-stool sample (but were not necessarily present pre-MS onset): for cases: headache (*n* = 1); atopic dermatitis/eczema (*n* = 1); long-term constipation (*n* = 1); history of seizures (*n* = 1); reactive airways disease and headache (*n* = 1); scoliosis (*n* = 1). For controls: kyphosis (*n* = 1); Raynaud phenomenon (*n* = 1)

For the Treg analyses, 24 samples were available (*n* = 9 controls; *n* = 15 MS cases [8/15 were DMD naïve]). For the intracellular cytokine analyses, 21 samples were available (*n* = 9 controls; *n* = 12 MS cases [6/12 were DMD naïve]).

### Comparison of cases and controls: gut microbiota and blood immune markers

Similar to that reported in our prior gut microbiota study [27] (from which the current cases and controls were a sub-set), there were no differences between cases (all cases as well as DMD exposed or naïve cases) and controls for any of the alpha diversity metrics (Additional file 1: eTable 2). While there were no significant differences when all cases were compared to controls for the immune markers (*p* > 0.05, Additional file 1: eTable 2), some differences depending on DMD exposure status were observed (Additional file 1: eTable 2). Hence, where possible, DMD exposure was considered in the analyses, either through model adjustment or a separate model developed.

### Microbiota diversity-immune associations

**Fig. 1:**
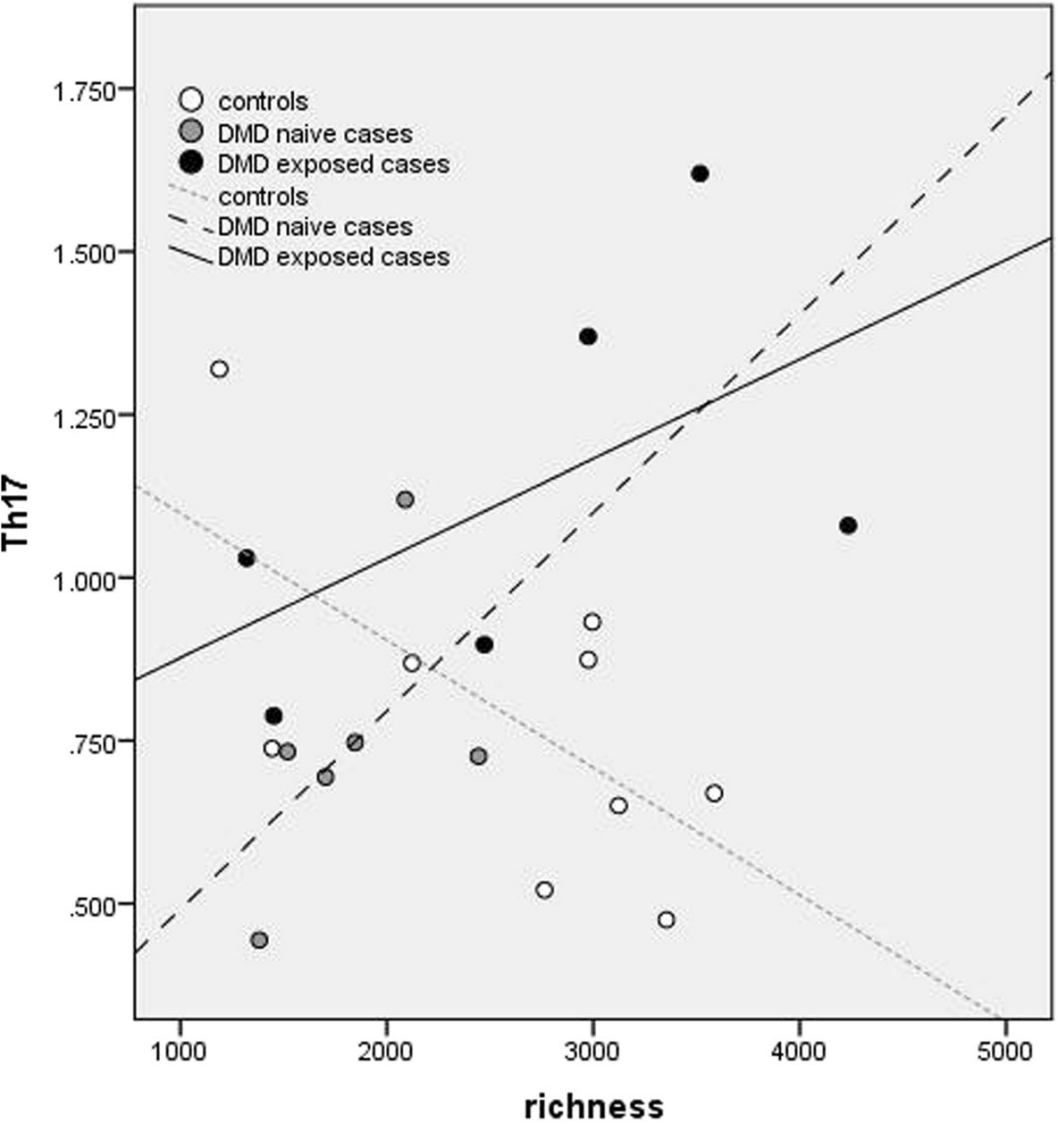
Divergence was observed for cases and controls in the associations between gut microbiota alpha diversity indices and peripheral blood immune markers: Gut diversity was negatively associated with Th17 in control children, but positively associated in cases. Richness is depicted for illustrative purposes. Pearson’s correlation coefficient and *p*-values for all children, controls, cases and by DMD exposure

While there were similarities for both cases and controls in the associations between their gut microbiota diversity metrics and host immunological markers, divergence was also apparent (Table 2, Fig. 1 and Additional file 1: eTable 3.1).

**Table 2:**
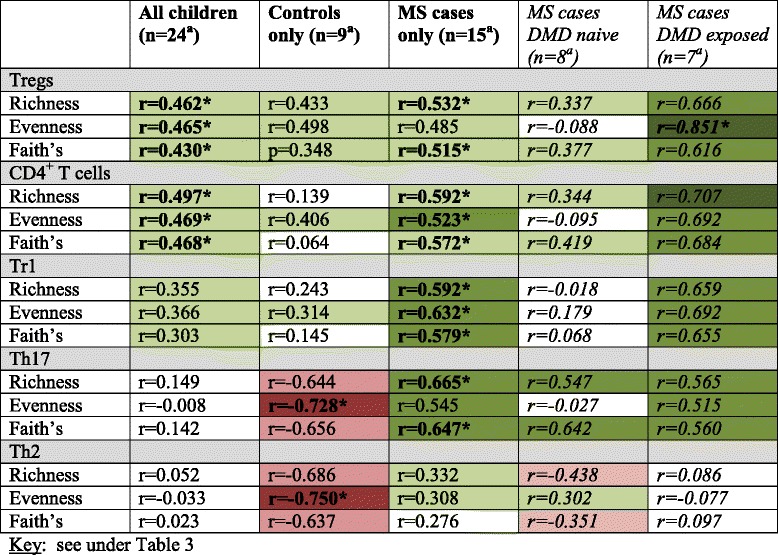
Associations between the gut microbiota alpha diversity metrics and peripheral blood immune markers and: all children, cases and controls

For controls, gut microbiota diversity was predominantly inversely associated with Th2 and Th17. However for cases, there were either no, or modest positive associations with Th2 and Th17, respectively (Table 2 and Fig. 1). After model adjustments, both immune markers remained significantly associated with evenness for the controls (age-adjusted *p* < 0.05, Additional file 1: eTable 3.1), indicating that gut microbiota dominated by specific taxa were associated with increases in these immune markers. For the cases, both richness and Faith’s diversity metric remained positively associated with Th17 (disease duration adjusted only, *p* = 0.008 and *p* = 0.013, respectively; *p* > 0.05 when age or DMD-adjusted, Additional file 1: eTable 3.1).

While positive associations were observed between the gut diversity metrics and Tregs, CD4^+^ T cells and Tr1 for both cases and controls, the strength and level of significance varied, often being more pronounced for the MS cases (Table 2). After model adjustments, significant associations remained with CD4^+^ T cells and Tr1 for cases only (the former when disease duration or DMD adjusted, the latter when age or disease duration but not DMD exposure adjusted). Tregs were the most strongly associated for the DMD exposed cases (evenness, *r* = 0.851, *p* = 0.015) which remained significant after age or disease duration adjustments (*p* = 0.034 and *p* = 0.017, respectively, Additional file 1: eTable 3.1).

For both cases and controls, no remarkable associations were observed between the gut microbiota diversity metrics and total T cells and Th1 (both *p* > 0.05; not all data shown).

### Microbiota phyla-immune associations

*Bacteroidetes* abundance was inversely correlated with several blood immune markers for cases and controls (Table 3). Both CD4^+^ T cells and Tregs remained independently associated, regardless of age or DMD exposure (all adjusted *p* < 0.05, Additional file 1: eTable 3.2). Cases in particular exhibited strong, negative associations between *Bacteroidetes* abundance and immune markers such as CD4^+^ T cells, Tregs and Th17 (r ranged from 0.613 to 0.719, all *p* < 0.02). All were independent of DMD exposure, and disease duration (*p* < 0.02, Additional file 1: eTable 3.2). Although only Tregs remained significant after age adjustment (*p* = 0.042).

**Table 3:**
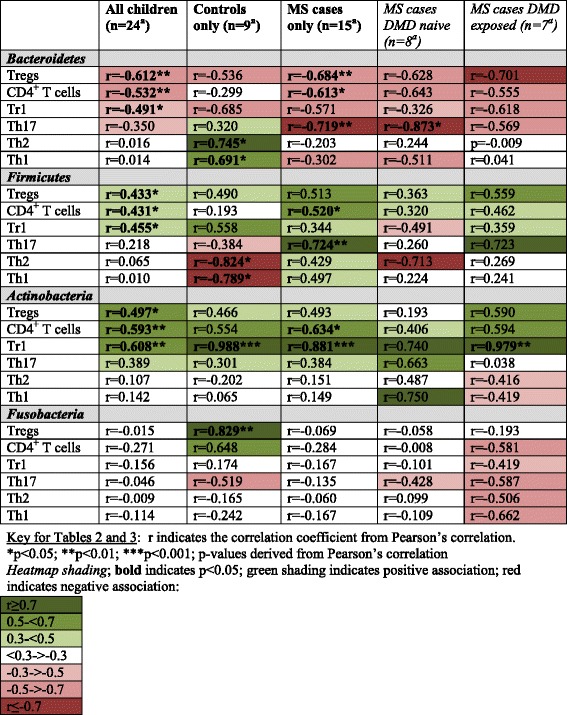
Association between the gut microbiota’s phylum-level abundance and peripheral blood immune markers

^a^immune markers were available for all 24 children, except as follows:: for Th17 and Th1 (*n* = 21 children; *n* = 9 controls, *n* = 12 cases [6 DMD exposed, 6 DMD naïve]); for Tr1 and Th2 (*n* = 20 children; *n* = 8 controls, *n* = 12 cases [6 DMD exposed, 6 DMD naïve])

No correction for multiple testing. Seven immune markers were assessed for correlation with three diversity metrics and 13 phyla, resulting in 7 × 3 = 21 tests (Table 2) and 7 × 13 = 91 tests (Table 3) respectively. Chance significance would be expected for 1 in 20 tests, i.e. approximately 1 test (diversity) and 4–5 tests (phylum-level)

Remaining blood immune markers (total T cells) and phyla (*Verrucomicrobia, Proteobacteria, Euryarchaeota, Tenericutes, Cyanobacteria, Deferribacteres, Synergistetes, TM7* and *Lentisphaerae*) are not shown here due to few significant findings and/or low abundances for the phyla. Except as follows

For all children: *Lentisphaerae* and Tregs (*r* = 0.427, *p* = 0.037); *TM7* and total T cells (*r* = −0.590, *p* = 0.002)

Cases: *Lentisphaerae* and Tr1 (*r* = 0.703, *p* = 0.011); *TM7* and total T cells (*r* = −0.628, *p* = 0.012)

In contrast, positive correlations (modest or strong) were observed for controls for Th1 and Th2 (*r* > 0.69, *p* = 0.034 and *p* = 0.039, Table 3). However, neither remained significant after age adjustment (*p* > 0.05, not shown).

*Actinobacteria* abundance was positively associated with several blood immune markers for both cases and controls. The strongest being with Tr1 and CD4^+^ T cells; both were independent of age or DMD (*p* < 0.005, Additional file 1: eTable 3.2). However, the abundance of *Fusobacteria* was positively associated with Tregs for controls only (age adjusted *p* = 0.009, Additional file 1: eTable 3.2), with no evidence of a relationship (positive or negative) for cases (Table 3).

The *Firmicutes*-immune associations were mixed (Table 3); divergence between cases and controls included strong positive association with Th17 for cases only (independent of disease duration or DMD exposure, both adjusted p-values were <0.05, but not age, *p* = 0.089). Conversely for controls (not cases), strong inverse associations with Th1 (*r* = −0.789, *p* = 0.011) were observed, remaining significant after age adjustments (*p* < 0.03, Additional file 1: eTable 3.2).

## Discussion

Associations were found between the composition of the gut microbiota and the host blood immune marker profiles in children with and without MS. While there were similarities for both cases and controls, there were also several divergent findings which were independent of DMD exposure. Further, these divergent microbiota-immune relationships were measurable very early in the MS disease course, as all pediatric cases were within 24 months of symptom onset. Disruption of microbiota-immune balance so early in MS is intriguing.

Gut microbiota evenness was strongly and inversely associated with Th17 and Th2 for the control children. However, there was either no or modest positive associations for the MS cases. While we were unable to find a similar study with which to compare our findings, a recent Canadian study demonstrated that diversity depleted uneven neonatal gut microbiota preceded later childhood-onset atopy and asthma risk [2]. In addition, mono-colonization i.e. an uneven community of the murine gut microbiota with segmented filamentous bacteria results in significant expansion of ileal lamina propria Th17 populations [15]. Our observations were consistent with these studies and suggest that uneven microbiota (which can indicate species overgrowth) in the pediatric gut is associated with increases in markers of Th17 and Th2 populations. Why this microbiota-immune relationship is reversed or lost in the MS children is of interest and may be due to the specific composition of gut microbial communities in MS versus control populations.

Nonetheless, there were also similarities for the cases and controls in that both groups exhibited positive associations between the gut microbiota diversity metrics and Tregs, CD4^+^ T cells and Tr1. Not all findings were independent of age which may be suggestive of an evolving microbiota-immune relationship as the child develops [16]. Also, although the direction of effect was similar for cases and controls, the strength of these associations were greater for the MS cases in a number of instances, particularly for the DMD exposed group. For instance, despite no significant differences between controls and cases (DMD exposed or naïve) for Tregs, this immune marker was strongly associated with diversity (evenness) for the DMD exposed cases (i.e. had a high r-value: *r* = 0.851, *p* = 0.015) which was independent of age or disease duration (*p* < 0.05).

### Microbiota phylum abundance-immune associations

There were several microbiota-immune associations measurable at the phylum-level which were consistent across both cases and controls. These included *Bacteroidetes* and *Actinobacteria* which represent two of the most common bacterial phyla in the human gut. The relative abundance of *Bacteroidetes* was negatively associated with both CD4^+^ T cells and Tregs, and strong positive associations were observed between *Actinobacteria* and CD4^+^ T cells and Tr1, all of which were independent of age or DMD exposure. These observations concur with the wider literature. For instance, *Bacteroidetes* includes species such as *Bacteroides fragilis*, well studied in animal models for their ability to induce CD4^+^ T cells [20, 21]. In addition, Tr1, i.e. IL-10 are reported as one of the main immunoregulatory factors required for immune tolerance of the intestinal microbiota [20], such that a strong relationship with one of the main gut phyla would not be unexpected.

However, divergences in the phylum-immune relationship were observed. Of interest were two associations only observed in the control children; *Fusobacteria* abundance exhibited a strong, positive association with the Tregs and the *Firmicutes* phylum was inversely associated with Th1 (*r* = 0.829 and *r* = −0.789, respectively, *p* < 0.02). Both were independent of age. Conversely, for cases, no strong relationships (either positive or negative) were observed between these phyla and immune markers. These differences are likely due to the specific composition of these phyla, each of which consists of hundreds of functionally distinct species and strains. For example members of *Clostridiales* Clade IV and XIV can induce Treg cells [3] while a related Firmicute, *Clostridium difficile* is a well described human pathogen. Findings highlight the need to study much larger cohorts, sufficiently powered to identify specific gut microbiome members that exhibit relationships with these important adaptive immune responses.

While little is known of the gut-related *Fusobacteria*-immune relationships, there is some evidence to suggest that dysregulation of Tregs may contribute to disease processes in MS, specifically an increased risk of relapse [25]. Loss of the *Fusobacteria*-immune relationship for cases may be clinically relevant and possibly linked to our previous observation that depletion of *Fusobacteria* in the gut microbiota of pediatric MS cases was associated with a higher risk (hazard) of relapse [26]. It would be of value for future studies to examine the gut-immune relationship in the context of MS disease activity (i.e., clinical relapses or MRI-related metrics).

### Possible mechanisms of action

While we modelled the gut microbiota as a predictor of host immune markers, the two are considered to have coevolved with likely a bi-directional relationship [18]. Although the potential mechanism(s) of action are not well understood [1], the gut microbiota are known to elicit Treg recruitment, regulate Th17 and Treg cell differentiation, which in the healthy gut promotes immune-microbiota homeostasis [19]. Metabolites produced by the gut microbiota such as short chain fatty acids (e.g., butyrate) have been shown to exert immunomodulatory affects, including down-regulation of pro-inflammatory mucosal Th2 responses [1, 10, 19, 28]. As suggested by our findings, and others, the effects of microbiota compositional changes are not exclusively local, but can influence the systemic immune response and, as others have shown, are associated with systemic inflammatory diseases distal to the gut, such as rheumatoid arthritis [19]. In the context of conditions such as inflammatory bowel disease, others have concluded that ‘dysregulated CD4 T cells responses to antigens of the microbiota leads to chronic, typically relapsing and remitting disease that reflects the immune system’s inability to eliminate the antigens that drive the abnormal response [20].’ This could apply to MS, also a predominantly relapsing-remitting disease. Evidence to suggest that gut microbiota can trigger non-gut related diseases includes the observation that *Clostridia sp., Candidatus arthromitus* or segmented filamentous bacteria in mice can cause inflammatory arthritis [19] and MS-like symptoms in EAE [17] mediated via Th17 production.

### Strengths and limitations

Limitations of this pilot study include the small sample size and no correction for multiple testing. Some findings would be expected by chance alone, such that we have specifically focused on those that were modest or strong and significant (absolute *r* > 0.5 and *p* < 0.05), appeared consistent across related biological markers and, wherever possible, developed models adjusting for potential confounders. In addition, we purposely focused on a high-level view of the gut microbiota, i.e., diversity and phylum-level abundance. Our findings require replication, but are also necessary to help guide and power future studies. Further exploration is needed, when the sample size permits, such as: to understand the specific functions being performed by the gut microbiota, or key members of the community present; to gain a better understanding of the influence of a child’s age on the microbiota-immune relationship; and to examine the influence of host genetics [11], diet, drug exposure and other effect modifiers.

Strengths include a well-phenotyped group of children with a rare but life changing chronic condition; less than 5 % of individuals with MS develop the disease and are diagnosed during the childhood years. All MS cases were examined and diagnosed by a pediatric MS neurologist and enrolled close to symptom onset with paired stool and blood samples collected within a short time of each other. Our study offers novel insights; the gut microbiota has not been well characterized in children (e.g. the Human Microbiome Project, HMP only included adults over 18 years [14]). Further, we are not aware of another study exploring the microbiota-immune relationship in MS (adults or children).

## Conclusions

Understanding the relationship between diseases considered immune-mediated, such as MS, and the gut microbiota, which has a central role in shaping the immune response, is fundamental [20]. We found associations between the gut microbiota composition and host blood immune markers which were divergent between cases and controls. Why the microbiota-immune balance appears disrupted in MS is intriguing and deserves further study. A better understanding and identification of key microbiota members involved may lead to novel opportunities to modulate immune dysregulation in diseases such as MS.

## Additional file

Additional file 1: Supplementary Methods (Appendix 1) and eTables 1 to 3.2 [23, 29, 30]. (DOCX 38 kb)

## Abbreviations

BMI: Body mass index
CD3 CD4: Cluster of differentiation (protein) 3, 4
DMD: Disease-modifying drug (DMD)
DNA: Deoxyribonucleic acid
EAE - EDSS: Expanded Disability Status Scale score
HMP: Human microbiome project
MRI: Magnetic resonance imaging
MS: Multiple sclerosis (MS)
OTU: Operational taxonomic unit
PBMC: Peripheral blood mononuclear cell
QIIME: Quantitative Insights Into Microbial Ecology
RNA: Ribonucleic acid
SD: Standard deviation
Th1 Th2: Type 1, Type 2T helper (cell)
Treg: T regulatory (cell)
UCSF: University of California San Francisco
