# Supplementary material for: Associations between the gut microbiota and host immune markers in pediatric multiple sclerosis and controls
Source: BMC Neurol. 2016 Sep 21;16:182. doi: 10.1186/s12883-016-0703-3 (PMC5031272; doi:10.1186/s12883-016-0703-3)
Supplement: Additional file 1: — Supplementary Methods (Appendix 1) and eTables 1 to 3.2 [23, 29, 30]. (DOCX 38 kb) [file 12883_2016_703_MOESM1_ESM.docx]

**Supplementary Methods (Appendix 1) and eTables 1 to 3 for online viewing only**

**Outline:**

**Appendix 1: Supplementary methods**

*1.1: Diversity metrics*

*1.2: Multivariable linear regression models*

**eTable 1 (1.1 and 1.2): Additional characteristics of the pediatric multiple sclerosis (MS) cases and controls**

*1.1: Diet, lifestyle and very early life exposures for the cases and controls*

*1.2: Clinical characteristics of the children with multiple sclerosis (cases)*

**eTable 2: Summary of the diversity metrics and immune markers for cases and controls, and by disease-modifying drug (DMD) exposure**

**eTable 3.1: Gut microbiota diversity metrics as independent predictors of peripheral blood immune markers: multivariable linear regression**

**eTable 3.2: Phylum-level gut microbiota abundances as independent predictors of blood immune markers: multivariable linear regression**

**Appendix 1: Supplementary methods**

*1.1: Diversity metrics*

Alpha diversity, as measured here, represents a global measure of the ‘within sample’ biodiversity of a given stool sample. Originally developed to describe ecological landscapes,[[30](#_ENREF_30)] this metric has been readily adapted to describe the ecology of the gut microbiota. Richness represented the number of unique taxa (species-level OTUs). Evenness indicated how evenly (or equally) distributed these taxa were. Faith’s phylogenic diversity metric[[8](#_ENREF_8)] takes into consideration the phylogenetic (i.e. based on the ‘evolutionary tree’) relationship between taxa for each sample. From the wider literature, a higher diversity (richness, evenness or Faith’s) is typically considered a better marker of the overall health of the microbiota. Conversely, a low diversity is generally considered unfavourable. Examples include disease such as inflammatory bowel disease and whereby presence of disease is associated with low gut microbiota diversity, relative to healthy controls.[[23](#_ENREF_23)]

*1.2: Multivariable linear regression models*

Selection of the gut microbiota and blood immune markers to include in multivariable linear regression models were based on the (univariate) Pearson’s correlation coefficients from Tables 2 and 3. Data distribution and the relationship between variables were initially assessed via histograms and scatter plots. Judicious selection of models and covariates were made, with the following principles in mind: i) if at least ‘modest’ and significant correlation was observed (absolute r>0.5 and p<0.05, from Pearson’s correlation), then a multivariable linear model was developed; ii) as only a limited number of covariates could be included in each model (using the ‘1 in 10’ rule of thumb, two covariates could be included when ‘all children’ were analysed, 1-2 covariates for the MS cases only and 1 covariate for the other subgroups (controls only or DMD naïve or exposed cases only).

The potential confounders selected for model adjustment were: age (continuous); case or control status; disease duration (continuous); DMD exposure (exposed or naïve); or MS/DMD exposed status (DMD exposed cases, DMD naïve cases or control). For the latter, as only continuous or binomial covariates can be include in the linear regression models, binary dummy variables were created for inclusion, derived from the three-level categorical variable ‘DMD exposed, DMD naïve or control.’

Based on the principles outlined above, for each gut microbiota/ immune marker linear regression analyses performed, model adjustments were made as follows: when all children were analysed, age (model 1), age and case or control (model 2) and MS/DMD exposed (model 3). When cases only were analysed, age (model 1), disease duration (model 2) and DMD exposure (model 3). When DMD naïve or DMD exposed cases only were analysed, age (model 1), disease duration (model 2). When controls only were analysed, age (model 1).

**eTables 1.1 and 1.2: Additional characteristics of the pediatric multiple sclerosis (MS) cases and controls**

***1.1: Diet, lifestyle and very early life exposures for the cases and controls***

| **Characteristic,** n (%) unless stated otherwise | **MS cases, n=15** | **Controls, n=9** | **Cases and controls, n=24** |
| --- | --- | --- | --- |
| ***Diet, lifestyle and very early life exposures*** | | |  |
| **Mode of delivery:** vaginal birth | 13 (87%) | 9 (100%) | 22 (92%) |
| Caesarean section | 2 (13%) | 0 | 2 (8%) |
| **Breastfed:** <12 months | 10 (67%) | 6 (67%) | 16 (67%) |
| ≥12 months | 5 (33%) | 3 (33%) | 8 (33%) |
| **Antibiotic exposure in 1^st^ year of life**: yes | 4 (27%) | 3 (33%) | 7 (30%) |
| No | 10 (67%) | 5 (56%) | 15 (63%) |
| Unknown | 1 (7%) | 1 (11%) | 2 (8%) |
| **Attended day-care (‘preschool’)**: yes | 11 (73%) | 7 (78%) | 18 (75%) |
| No | 2 (13%) | 1 (11%) | 3 (13%) |
| Unknown | 2 (13%) | 1 (11%) | 3 (13%) |
| **BMI** [1]: crude mean (SD; range) | 22.2 (SD:6.21; range:15.1-35.3) | 20.9 (SD:4.89; range:15.1-28.7) [missing=1] | 21.8 (SD:5.70; range:15.1-35.3) [missing=1] |
| Healthy or underweight(<85^th^ percentile) | 9 (60%) | 6 (75%) | 15 (65%) |
| Overweight or obese (≥85^th^ percentile) | 6 (40%) | 2 (25%) [missing=1] | 8 (35%) [missing=1] |
| **Fat/fibre diet groups** [2]:  High fat, and low or very fibre – yes | 3 (20%) | 2 (22%) | 5 (21%) |
| No | 12 (80%) | 7 (78%) | 19 (79%) |

***1.2: Clinical characteristics of the children with multiple sclerosis (cases)***

| **Characteristic,** n (%) unless stated otherwise | **N=15** |
| --- | --- |
| **Age at MS symptom onset**, years: mean (SD; range) | 11.5 years (SD=4.84; 4-17) |
| **Disease duration at stool collection** [3], months: mean (SD; range) | 10.0 months  (SD=6.42; 2.3-23.1) |
| **Annualized relapse rate pre-stool sample** [excl. onset attack], mean (SD; range) [4] | 0.91 (SD=0.951; range: 0-2.9) |
| **Time since last relapse** (in relation to stool sample; onset attack considered): days: mean (SD; range) | 181.1 days (SD=142.17; 4 to 489 days) |
| **Time since last relapse** (in relation to stool sample; onset attack considered): n (%): <90 days | 6 (40%) |
| 90-<200 days | 4 (27%) |
| 200+days | 5 (33%) |
| **Disability level - EDSS** at enrolment, median (range) | 2.0 (0-4.0) |
| 0-<2.0 | 5 |
| 2.0-<3.0 | 7 |
| 3.0+ | 3 |
| **Disease-modifying drug exposure status** [5]: DMD naïve | 8 (53%) |
| DMD exposed | 7 (47%) |
| **Corticosteroids – systemic** [6]: No | 10 (67%) |
| Yes | 5 (33%) |

Key: SD=standard deviation; excl.=excluding; EDSS=Expanded Disability Status Scale score; DMD=disease-modifying drug

[1] Body Mass Index (BMI)=height(kg)/weight(m)sq, with age and sex-specific percentiles (standardized against age and sex growth charts from the Center for Disease Control[[29](#_ENREF_29)] to obtain a percentile ranking). One individual (case) was below the 5^th^ percentile; 3/15 cases and 1/9 control were ≥95^th^ percentile (obese)

[2] Recent diet derived from the Block Kids Food Screener (NutritionQuest©), recorded over one week (as described previously[[27](#_ENREF_27)]).

[3] disease duration: time from symptom onset to stool collection

[4] relapse rate = relapse count/disease duration; note however that while a major strength of the study, the disease duration was low; i.e. those cases with the shortest disease durations pre-stool sample can influence the apparent relapse rates as follows: a very short disease duration implies less opportunity for a relapse to occur, therefore often the relapse rate was ‘zero.’ However, if a relapse did occur, the resulting relapse rate can appear high (inflated) because of the small denominator (disease duration)

[5] ‘DMD naïve’ indicates never exposed pre-stool sample. ‘DMD exposed’ indicates ever exposed pre-stool sample; all were also currently exposed (at the time of stool collection) as follows: beta-interferon (n=2); glatiramer acetate (n=5). No child had switched or stopped a DMD (although one child had previously been exposed to plasma exchange before taking glatiramer acetate).

[6] within the 2 months prior to stool sample collection

**eTable 2: Summary of the diversity metrics and immune markers for cases and controls, and by disease-modifying drug (DMD) exposure**

|  | **Controls,** n=9 [A] | **All MS cases**  (DMD naïve and exposed combined),  n=15 | *DMD naïve cases, n=8*  [B] | *DMD exposed cases,*  *n=7* [C] | Three-way group comparison: Controls vs DMD naïve cases vs DMD exposed cases  [A vs. B vs C]‡ |
| --- | --- | --- | --- | --- | --- |
| ***Gut microbiota diversity metrics***, median (quartiles) | | | | |  |
| Richness | 1783  (2976, 3239) | 1452  (2089, 2975) | 1416  (1775, 2355) | 1452  (2472, 3517) | ns |
| Evenness | 0.312 (0.346, 0.386) | 0.282  (0.319, 0.378) | 0.274  (0.309, 0.339) | 0.296  (0.366, 0.393) | ns |
| Faiths | 69.0  (105.4, 123.9) | 58.1  (83.9, 107.7) | 58.2  (71.8, 96.6) | 54.5  (95.0, 128.8) | ns |
| ***Host Immune blood markers***, median (quartiles) | | | | |  |
| Total T cells | 76.4  (70.5, 79.6) | 75.1  (69.6, 78.1) | 73.5  (60.5, 76.7) | 77.0  (75.1, 78.8) | ns |
| CD4^+^ T cells | 53.6  (51.8, 60.2) | 53.4  (47.0, 58.6) | 51.9  (47.4, 54.0) | 58.6  (46.9, 65.3) | ns |
| Tregs | 0.90  (0.81, 1.42) | 1.18 (1.00, 1.47) | 1.15  (0.80, 1.42) | 1.18  (1.01, 1.54) | ns |
| Th1 | 7.2 (4.9, 12.5) | 7.4 (5.1, 17.9)  Missing, n=3 | 5.3 (3.1, 7.7)  Missing, n=2 | 16.4 (6.9, 21.7)  Missing, n=1 | ns |
| Th17 | 0.74 (0.59, 0.90) | 0.84 (0.73, 1.11)  Missing, n=3 | 0.73 (0.63, 0.84)  Missing, n=2 | 1.06 (0.87, 1.43)  Missing, n=1 | **p=0.036** |
| Th2 | 1.8 (1.0, 2.0)  Missing, n=1 | 1.9 (1.3, 4.1)  Missing, n=3 | 1.3 (0.3, 1.9)  Missing, n=2 | 3.6 (1.8, 5.1)  Missing, n=1 | **p=0.036** |
| Tr1 | 0.636 (0.369, 1.008)  Missing, n=1 | 0.234 (0.178, 0.554)  Missing, n=3 | 0.187 (0.000, 0.543)  Missing, n=2 | 0.334 (0.180, 0.821)  Missing, n=1 | ns |

Key: ‡p-vales derived from the Kruskal-Wallis test; ns=not significant, i.e. p<0.05. No adjustments made for multiple comparisons

Additional pairwise comparisons were performed using the Mann-Whitney U-test as follows: i) all cases vs. controls; ii) DMD naïve vs. exposed cases; iii) DMD naïve cases vs. controls. All p>0.05, except for some of the immune marker metrics only, as follows:

i) DMD naïve cases vs. controls: Tr1 p=0.039 (higher in controls);

ii) DMD naïve vs. exposed cases: Th1; Th17; Th2 (all were higher in DMD exposed, p=0.030, p=0.025, p=0.037, p=0.025, respectively). While the latter finding (Th2) concurs with the adult MS literature, the former observations (for Th1, Th17) were unexpected. However, the pediatric literature is limited. We also did not have access to pre-treatment immune markers in these individuals such that we cannot determine if these individuals had particularly high levels pre-treatment.

**eTable 3.1: Gut microbiota diversity metrics as independent predictors of peripheral blood immune markers: multivariable linear regression**

|  | **Beta coefficient (95%CI)** | **p-value** |
| --- | --- | --- |
| **Controls only, n=9** | **Age adjusted** |  |
| **Evenness** and **Th17** | **-2.9 (95%CI: -5.7 to -0.2)** | **p=0.039** |
| **Evenness** and **Th2** | **-13.1 (95%CI: -25.7 to -0.6)** | **p=0.044** |
| **Cases only, n=15** | **[1] Age adjusted**  **[2] Disease duration adjusted**  **[3] DMD exposure adjusted** |  |
| **Richness** and **Tr1** | **[1] 2.7e-4 (95%CI: 0.08-10)** | **p=0.045** |
|  | **[2] 2.5e-4 (95%CI: 0.17-** **4.7)** | **p=0.038** |
|  | [3] 1.8e-4 (95%CI: -0.51 to 4.2) | p=0.110 |
| **Evenness** and **Tr1** | **[1] 4.1 (95%CI: 0.1-8.1)** | **p=0.047** |
|  | **[2] 5.6 (95%CI: 1.4-9.8)** | **p=0.015** |
|  | [3] 3.8 (95%CI: -0.44 to 8.0) | P=0.073 |
| **Faith’s** and **Tr1** | **[1] 8.0e-3 (95%CI:** **0.06-16.0)** | **p=0.049** |
|  | **[2] 7.0e-3 (95%CI: 0.028-13.0)** | **p=0.049** |
|  | [3] 5.0e-3 (95%CI: -1.4 to 11.4) | p=0.110 |
| **Richness** and **CD4^+^ T cells** | [1] 0.004(95%CI:-0.001to 0.008) | p=0.081 |
|  | **[2] 0.004 (95%CI:0.001to 0.008)** | **p=0.027** |
|  | **[3] 0.004 (95%CI: 0.0001- 0.008)** | **p=0.043** |
| **Faith’s** and **CD4^+^** **T cells** | [1] 0.1 (95%CI: -0.02 to 0.2) | p=0.101 |
|  | **[2] 0.12 (95%CI: 0.01-0.23)** | **p=0.035** |
|  | **[3] 0.11 (95%CI: 0.006-0.21)** | **P=0.039** |
| **Richness** and **Th17** | [1] 1.23e-4 (95%CI: -0.1.1 to 3.5) | p=0.258 |
|  | **[2]** **3.0e-4 (95%CI: 1.0-** **5.0)** | **p=0.008** |
|  | [3] 1.7 e-4 (95%CI: -0.3 to 3.7) | p=0.087 |
| **Faith’s** and **Th17** | [1] 0.003 (95%CI: -0.004 to 0.010) | p=0.388 |
|  | **[2] 0.008 (95%CI:0.002-0.014)** | **p=0.013** |
|  | [3] 0.005 (95%CI: -0.0005 to 0.0101) | p=0.069 |
| **DMD exposed cases only, n=7** | **[1] Age adjusted**  **[2] Disease duration adjusted** |  |
| **Evenness** and **Tregs** | **[1] 7.3 (95%CI: 0.9-13.7)** | **p=0.034** |
|  | **[2] 0.04 (95%CI:** **-0.02 to 0.10)** | **p=0.017** |

**Key**: see under eTable 3.2

**eTable 3.2: Phylum-level gut microbiota abundances as independent predictors of blood immune markers: multivariable linear regression**

|  | **Beta coefficient (95%CI)** | **p-value** |
| --- | --- | --- |
| **Both cases and controls, n=24** | **[1] Age**  **[2] Age and case or control adjusted**  **[3] MS/DMD exposed adjusted‡ (DMD exposed cases, DMD naïve cases or control)** |  |
| ***Bacteroidetes*** and Tregs | **[1] -4.9e-6 (95%CI:** **-8.0 to -2.0)** | **p=0.007** |
|  | **[2] -4.8e-6 (95%CI: -8.0 to -1.0)** | **p=0.007** |
|  | **[3] -5.2e-6 (95%CI: -8.0 to -2.0)** | **p=0.002** |
| ***Bacteroidetes*** and CD4^+^ T cells | **[1] -7.6e-5 (95%CI: -14.8 to -0.5)** | **p=0.038** |
|  | **[2] -7.7e-5 (95%CI: -15.0 to -0.3)** | **p=0.042** |
|  | **[3] -7.6e-5 (95%CI: -14.0 to -1.3)** | **p=0.020** |
| ***Actinobacteria*** and CD4^+^ T cells | **[1]** **3.3e-4 (95%CI: 1.1-1.1)** | **p=0.005** |
|  | **[2] 3.4e-4 (95%CI: 1.2-5.6)** | **p=0.004** |
|  | **[3] 3.3e-4 (95%CI: 1.0-5.7)** | **p=0.008** |
| ***Actinobacteria*** and Tr1 | **[1] 3.1e-5 (95%CI: 0.9-5.3)** | **p=0009** |
|  | **[2] 3.7e-5 (95%CI: 0.19-5.5)** | **p<0.0001** |
|  | **[3] 4.1e-5 (95%CI: 2.3-6.0)** | **p=0.0002** |
| **Controls only, n=9** | **Age adjusted** |  |
| ***Firmicutes*** and Th2 | **-2.5e-5 (95%CI: -4.5 to 0.5)** | **p=0.023** |
| ***Firmicutes*** and Th1 | **-11.6e-5 (95%CI: -20.8 to -2.5)** | **p=0.021** |
| ***Fusobacteria*** and Tregs | **0.32 (95%CI: 0.11-0.53)** | **p=0.009** |
| **Cases only, n=15** | **[1] Age adjusted**  **[2] Disease duration adjusted**  **[3] DMD adjusted** |  |
| ***Bacteroidetes*** and Tregs | **[1] -0.5e-6 (95%CI: -10 to -0.2)** | **p=0.042** |
|  | **[2]** **-5.7e-6 (95%CI: -9.0 to -2.0)** | **p=0.006** |
|  | **[3] -5.7e-6 (95%CI: -10.0 to -2.0)** | **p=0.009** |
| ***Bacteroidetes*** and CD4^+^ T cells | [1] -9.5e-5 (95%CI: -20.7 to 1.7) | p=0.088 |
|  | **[2] -1.12e-4 (95%CI: -2.01 to -0.22)** | **p=0.019** |
|  | **[3] -9.7e-5 (95%CI: -18.6 to -0.9)** | **p=0.034** |
| ***Bacteroidetes*** and Th17 | [1] -3.0e-6 (95%CI: -7.0 to 1.0) | p=0.154 |
|  | **[2]** **-4.9e-6 (95%CI: -9.0 to -1.0)** | **p=0.013** |
|  | [**3] -3.9e-6 (95%CI: -7.0 to -0.7)** | **p=0.021** |
| ***Actinobacteria*** and CD4^+^ T cells | **[1] 3.5e-4 (95CI: 0.6-10)** | **p=0.023** |
|  | **[2] 3.9e-4 (95%CI: 0.88-7.0)** | **p=0.016** |
|  | **[3] 3.5e-4 (95%CI: 0.12-6.8)** | **p=0.043** |
| ***Firmicutes*** and Th17 | [1] 4.3e-6 (95%CI: -0.8- 9.0) | p=0.089 |
|  | **[2] 8.7e-6 (95%CI: 4.0-13.0)** | **p=0.002** |
|  | **[3] 5.1e-6 (95%CI: 0.1-10.0)** | **p=0.046** |

**Key**: DMD=disease-modifying drug

Where ‘e-5’ is shown, the corresponding 95%CIs are also shown as e-5; e-5 represents x10^-5^

‡ Age and MS/DMD exposed adjusted - three binary dummy variables were included in each model, derived from the three-level categorical variable ‘DMD exposed, DMD naïve or control’

[3] DMD adjusted; modelled as ‘DMD naïve vs. DMD exposed’

The beta coefficients represent changes in the immune marker for every unit increase in the gut microbiota measure

Selection of gut microbiota and blood immune markers was based on the univariate correlation coefficients from Tables 2 and 3. If (absolute) r>0.5 and p<0.05 (from Pearson’s correlation), see Appendix 1.2 for further details. Only findings (beta and 95%CIs) which reached significance after covariate adjustment (p<0.05) in at least one of the models are shown.

None of the covariates used to adjust models remained as significant independent predictors (p>0.05).

**References related to Supplementary files**

(Please see under main manuscript references)
